# Occupational Priorities of People on Hemodialysis Who Participated in Energy Management Education

**DOI:** 10.1177/00084174241271205

**Published:** 2024-08-07

**Authors:** Janine Farragher, Jane A. Davis, Chandra Thomas, Pietro Ravani, Braden Manns, Meghan J. Elliott, Brenda R. Hemmelgarn

**Keywords:** Dialysis, Energy management, Fatigue, Kidney disease, Occupational participation, Dialyse, fatigue, gestion de l'énergie, maladie rénale, participation occupationnelle

## Abstract

**Background.** People with kidney failure who undergo hemodialysis treatment and experience chronic fatigue identify negative effects on occupational performance and participation as a key aspect of their illness experience. **Purpose.** To describe the occupational performance and participation problems of people treated with hemodialysis who live with debilitating fatigue. **Method.** Fifteen participants, who were randomized to participate in an energy management intervention as part of a randomized controlled trial, completed two occupation-based assessments at baseline and chose three priority occupational performance or participation problems to address as goals during the intervention. Results were analyzed using descriptive statistics (counts and percentages). **Findings.** Fifteen participants (mean age 60, 53% male) completed the occupation-based assessments. Participants stated that they wanted or needed more energy for a median of 22 of 55 occupations. Going out for food/drinks (*n* = 11), going to a movie/concert/performance (*n* = 10), and food preparation/clean-up (*n* = 10) were the top occupations for which participants required more energy. Prioritized occupational performance and participation problems most often fell within the household management (14 goals), self-care (6 goals), and hobbies (5 goals) domains. **Conclusion.** Occupational performance and participation problems are extensive among people treated with hemodialysis who live with debilitating fatigue. There is a clear need for occupation-based interventions that optimize occupational performance and participation in this population.

## Résumé

**Description.** Les personnes atteintes d'insuffisance rénale qui subissent un traitement d'hémodialyse et qui souffrent de fatigue chronique identifient les effets négatifs sur la participation et le rendement occupationnels comme des aspects clés de leur expérience de la maladie. **But.** Décrire les problèmes de participation et de rendement occupationnels des personnes traitées par hémodialyse qui vivent avec une fatigue débilitante. **Méthodologie.** Quinze personnes, qui ont été randomisées pour participer à une intervention de gestion de l'énergie dans le cadre d'un essai contrôlé randomisé, ont effectué deux évaluations fondées sur l'occupation au départ, et ont choisi trois problèmes prioritaires en matière de participation ou de rendement occupationnels à aborder comme objectifs au cours de l'intervention. Les résultats ont été analysés à l'aide de statistiques descriptives (nombres et pourcentages). **Résultats.** Quinze participants (âge moyen de 60 ans, 53 % d'hommes) ont réalisé les évaluations fondées sur l'occupation. Les participants ont déclaré qu'ils voulaient ou avaient besoin de plus d'énergie pour une médiane de 22 occupations sur 55. Sortir manger/boire (n = 11), assister à un film/concert/spectacle (n = 10) et préparer les repas/faire le ménage (n = 10) étaient les principales occupations pour lesquelles les participants avaient besoin de plus d'énergie. Les problèmes de participation et de rendement occupationnels prioritaires relevaient le plus souvent des domaines de la gestion du ménage (14 objectifs), des autosoins (6 objectifs) et des loisirs (5 objectifs). **Conclusion.** Les problèmes de participation et de rendement occupationnels sont nombreux chez les personnes traitées par hémodialyse qui vivent avec une fatigue débilitante. Il existe un besoin évident d'interventions fondées sur l'occupation qui optimisent la participation et le rendement occupationnels au sein de cette population.

## Introduction

There are approximately 30,000 people in Canada who undergo maintenance hemodialysis therapy for kidney failure (Canadian Institute for Health Information, 2023). The ability to perform and participate in valued occupations can be greatly impacted by the symptoms of kidney failure and the arduous demands of hemodialysis treatment (Altintepe et al., 2006; Cook & Jassal, 2008; Jassal et al., 2016; McAdams-DeMarco et al., 2013), which requires three 4-hr treatment sessions every week. Historically, occupational performance and participation have been underrecognized as priority outcomes in kidney disease care (Johansen, 2019), which has resulted in limited access to rehabilitative services, outside of acute inpatient settings, to support occupational performance and participation as people attempt to cope with kidney failure and dialysis treatment (Jassal & Watson, 2010; Johansen, 2019; Matsuzawa & Kakita, 2022). However, the importance of occupational performance and participation has recently been highlighted in a series of research priority-setting exercises conducted with people who have kidney disease, their care partners, and other stakeholders across 63 countries. In this exercise, occupational performance and participation (collectively termed “life participation”) were chosen as one of the selected number of core health outcomes that kidney disease stakeholders wish to be captured in all future clinical trials (Manera et al., 2020a; Tong et al., 2017b; 2017c). This finding has provided a strong rationale to gain further insight into the occupational problems and priorities of people with kidney disease on dialysis treatment, so that supportive interventions can be developed and introduced into clinical practice.

Kidney failure and dialysis treatment appear to have a profound effect on occupational performance and participation across multiple occupational domains. In a global study involving 7,226 people on hemodialysis treatment, only one-third of participants were able to perform all of their routine self-care occupations (e.g., bathing, dressing, and grocery shopping) without assistance (Jassal et al., 2016). Unemployment among working-age adults on hemodialysis has been estimated at more than 50% (Brown et al., 2021), while people on hemodialysis frequently describe limitations in their ability to socialize, travel, and pursue valued hobbies (Clarkson & Robinson, 2010; Jacobson et al., 2019; Ju et al., 2018a, 2018b; Lee et al., 2007). These data reflect the broader experience of living with a complex chronic disease, where the impact on life roles and responsibilities is a key aspect of the illness experience (Lorig & Holman, 2003). In kidney failure, a population typically comprised of older adults, occupational performance, and participation are likely affected by several disease-related complications including muscle atrophy (Johansen et al., 2003), numerous symptoms (Weisbord et al., 2005), cognitive dysfunction (Murray et al., 2006; O’Lone et al., 2016), and mood disorders (Khan et al., 2019; Palmer et al., 2013). Among these complications, fatigue appears to be a particularly important mediator of occupational performance and participation. For example, people on hemodialysis have previously detailed a grueling cycle of fatigue that fluctuates with their hemodialysis treatment schedule, and a frequent need to rest on both dialysis and nondialysis days (Jacobson et al., 2019). They have spoken of sacrificing valued occupations to reserve energy for mandatory health management and family priorities (Jacobson et al., 2019), and an inability to adequately fulfill spousal, parental, or other relationship roles (Clarkson & Robinson, 2010; Jacobson et al., 2019). Occupational therapists could play a key role in mitigating the effects of fatigue and other chronic disease complications on the most valued occupations of these patient groups. The objective of this study was therefore to explore the top occupational problems and priorities of people with kidney failure on hemodialysis who live with debilitating fatigue, with a long-term goal of informing interventions to address their top areas of concern.

## Method

### Study Design

The data from this descriptive cross-sectional analysis were collected as part of a pilot randomized controlled trial (RCT) conducted in Calgary, Canada (Farragher et al., 2022). Fifteen participants on hemodialysis with debilitating fatigue, who were randomized to undergo an energy management education program, completed extra pre-intervention baseline assessments that captured their top occupational performance and participation problems and priorities. The study was approved by the University of Calgary's Conjoint Health Research Ethics Board and registered at ClinicalTrials.gov (#NCT03825770).

### Participants

Participants were recruited from six dialysis units in Calgary, Canada by a member of our research team. People were approached to participate if they were aged ≥18 years; on hemodialysis for ≥3 months; medically and cognitively stable (i.e., able to provide informed consent); and scored a mean of ≥4 on items 5, 7, 8, and 9 from the Fatigue Severity Scale (i.e., the questions that ask about disabling impacts of fatigue). Prospective participants were excluded if they were going to switch dialysis centres or modalities or undergo renal transplantation within 6 months of the time of recruitment; if they had inadequate written and verbal English comprehension for study activities; if they resided in a nursing home facility; or if they had vision loss preventing them from engaging with the study materials. We obtained written informed consent from each participant prior to initiating any study activities.

### Data Collection

The energy management program (i.e., the “PEP” program) tested in the RCT combined background information on energy management strategies with training in the Cognitive Orientation (CO) to daily Occupational Performance approach. For the CO-occupational problems (OP)-based portion of the program, participants were asked to work on three occupational problems that (1) could be improved using energy management strategies and (2) could feasibly be addressed within the 6-week duration of the program. We identified these relevant occupational performance and participation problems and priorities of study participants by administering the “Energy Inventory” card-sorting activity. The “Energy Inventory” was adapted from the Activity Card Sort (ACS; Baum & Edwards, 2001), a validated assessment of an older adult's participation in instrumental, social, and leisure activities that asks patients to sort 80 occupations (e.g., food preparation; visiting family/friends) into participation categories (Baum & Edwards, 2001). For the Energy Inventory exercise, the 80 life activities from the ACS were reduced to 55 to improve feasibility, using a systematic selection and combination process that took into account the importance rank of the activity from the original paper, and the relevance of the activity to current-day life (Farragher et al., 2020a, 2020b). Participants were asked to sort these 55 occupations into three categories: (1) I have enough energy for it, (2) I want or need more energy for it, and (3) I don’t do it and don’t want/need to. They were then asked to identify three priority OPs from their “I want or need more energy for it” list that they wished to address during the energy management education program, with the Canadian Occupational Performance Measure (COPM; Law et al., 1990) used to guide the discussion and prioritization process. In keeping with the COPM, participants were also asked to rate the importance of each selected OP on a Likert-scale of 1 to 10, with 1 representing the least importance and 10 representing the greatest importance.

### Data Analysis

We calculated the median number of occupations participants sorted into the “I want/need more energy for it” category, and counted the number of times each occupation was placed in into the “I want/need more energy for it” category. We also counted the number of times each occupation was selected as a top-three priority OP. We categorized each OP into 15 activity and participation domains derived from the World Health Organization's International Classification of Function (ICF) (World Health Organization, 2019), and counted the number of top OPs chosen from each domain. Finally, we calculated the median importance rating participants assigned to their top three OPs on the COPM, and the median importance rating assigned to all of the OPs within each ICF activity and participation domain.

## Results

### Participants

Baseline characteristics of study participants are described in Table 1. The mean age of participants was 60 years, 53% were male, and 40% had diabetes. Sixty seven percent were living independently with no support needed for basic activities of daily living, and 27% were employed. Thirty-three percent screened positive for cognitive impairment on the MiniCog cognitive screen, and 47% screened positively for depression according to the Personal Health Questionnaire 2 depression screening tool.

**Table 1:** Participant Characteristics (*n* = 15).

Participant characteristic	*n* (%)
Age (mean, SD)	60.0, 15.1
Male	8 (53)
Independent living	13 (86)
Lives alone	4 (27)
Married	7 (46)
Employed	4 (27)
Dialysis vintage (median, IQR)	4.0 (1.7–9.5)
Diabetes	6 (40)
Depression	6 (40)
Coronary artery disease	4 (27)
Congestive heart failure	5 (33)
Cerebrovascular disease	0 (0)
Alzheimer's disease	1 (7)
Multiple sclerosis	1 (7)
Chronic obstructive pulmonary disease	0 (0)
Cancer	2 (13)
Baseline serum hemoglobin (g/L) (mean, SD)	95.0, 23.3
Baseline serum albumin (g/L) (mean, SD)	37.2, 15.0
Activities of daily living dependence	5 (33)
MiniCog impaired	5 (33)
Personal Health Questionnaire 2 impaired	7 (47)

Abbreviation: IQR=interquartile range.

### Occupational Priorities

Of the 55 occupations assessed in the Energy Inventory activity, participants wanted or needed more energy for a median of 22 occupations (interquartile range [IQR] 12.5, 23.5). Occupations that fell within the ICF categories of arts and culture, socializing, using transportation, and work/employment were most often selected as those for which participants needed more energy. The most common specific occupations for which the participants reported needing more energy were going out for food/drinks (*n* = 11), going to a movie/concert/performance (*n* = 10), food preparation/clean-up (*n* = 10), and deliberate exercising (*n* = 9) (see Table 2).

**Table 2:** Life Participation Priorities and Goals of Intervention Participants.

ICF category	Life activity	Number of participants who wanted/needed more energy to do it	Number of participants who selected it as a goal
Arts & culture	Going to a movie/concert/performance	●●●●●●●●●●	
	Visiting public attractions	●●●●●●●	●
Assisting others	Caregiving (not children)	●●●●	
	Child care	●●	
	Caring for pets/animals	●	●
Crafts	Crafts/handwork/creative writing	●●●●●●●	
Education	Studying & educational activities	●●●●●	
Hobbies	Cooking as a hobby	●●●●●●●●●	
	Puzzles & brain games	●●●●●●●	
	Restful outdoor activities	●●●●●●	●
	Shopping for enjoyment	●●●●●●	
	Table games	●●●●●●	
	Caring for plants	●●●●●	●●
	Interior decorating	●●●●●	
	Driving for leisure	●●●●	
	Photography	●●●●	
	Reading books/magazines/other	●●●	
	Yard or outdoor activities	●●●	●●
	Playing/writing music	●●	
	Listening to music	●	
Household tasks	Food preparation/clean-up	●●●●●●●●●●	●●●●●●
	Laundry/clothes care	●●●●●●●	●●
	Heavy household chores	●●●●●●	●●
	Home/yard maintenance	●●●●●●	●
	Light household chores	●●●●●●	●●●
	Money management	●●●	
	Vehicle maintenance/repairs		
Intimate relationships	Romantic activities	●●●●●●	
Recreation & leisure	Going out for food/drinks	●●●●●●●●●●	●●
	Travelling	●●●●●●●	●●
	Special interest group/club/course	●●●●●●	
	Using the internet	●●●●●	
	Video/computer games	●●●	
	Thinking/reflecting/resting	●●	
	Watching TV/movies/videos	●●	
Religion/spirituality	Religious/spiritual activities	●●●●●	●
Self-care	Health-related activities	●●●●●●	●●
	Washing/grooming	●●●●●●	
	Bathing/showering	●●●●●	●●
	Beauty care	●●●●●	
	Getting dressed/undressed	●●●●●	●
	Using the bathroom	●●●●●	
Shopping	Shopping for goods	●●●●●●●●	●●
Socializing	Entertaining at home	●●●●●●●●●	●
	Socializing at a party/club	●●●●●●●●	
	Visiting family/friends/neighbours	●●●●●●●	●
	Communicating via phone/text/internet/letter	●●●	
Sports	Deliberate exercising	●●●●●●●●●●	●
	Dancing	●●●●●	
	Individual sports	●●●●	●
	Pilates/yoga/tai-chi	●●	
	Water activities	●●	●
	Team sports	●	
Using transportation	Driving/using public transportation	●●●●●●●●	
Work & employment	Employment or volunteer activities	●●●●●●●	●

*Note*. ● = one participant.

The 15 participants selected a total of 42 occupational performance and participation problems to work on in the energy management program (see Table 2). The most common ICF category from which their OPs were selected were household tasks (16 OPs), followed by hobbies (6 OPs) and self-care (5 OPs). The median importance rating participants assigned to their OPs was an 8 out of 10; participants assigned the highest median importance to OPs within the activity and participation domains of employment (10/10; *n* = 1), hobbies (9.5/10), socializing (9.5/10), household tasks (9/10), and assisting others (9/10).

## Discussion

In this study, we explored the occupational performance and participation problems and priorities of people on hemodialysis who lived with debilitating fatigue. We found that participants reported widespread impacts on their occupational performance and participation, feeling that they had insufficient energy to perform or participate in a large number of occupations to their satisfaction. Participants reported negative effects across the spectrum of ICF activity and participation categories, frequently affecting multiple self-care, work, and leisure occupations. While household responsibilities such as cooking, laundry, and household chores emerged as the top occupational priorities, hobbies, and self-care occupations were also highly prioritized by our participants. These results underscore the need to identify interventions that can support people with kidney failure in maximizing their occupational performance and participation, and ensure that assessments and interventions used with people on hemodialysis address a diverse range of occupational performance and participation problems and priorities.

Our finding that participants’ occupations were so extensively impacted by fatigue is consistent with previous literature describing the lived experience of kidney failure and other complex chronic diseases (Jacobson et al., 2019; Ju et al., 2018a, 2018b). People on hemodialysis have previously described being deprived of time; losing the ability to work and provide for their family; feeling that they were failing as a parent; having an inability to do activities for enjoyment; and failing to meet others’ role expectations of them (Jacobson et al., 2019). Our finding that participants felt a median of 22 valued occupations were negatively affected quantifies this experience of occupational disruption and dysfunction, and underscores the urgent need to implement interventions that can support occupational performance and participation in this patient group. Traditionally, interventions for the chronic kidney disease population have been predominantly medical, failing to draw on the diverse range of evidence-based strategies used by occupational therapists to address physical, cognitive, psychosocial, and environmental barriers to occupational performance and participation. With respect to fatigue, the majority of nonpharmacological interventions have used exercise training as the primary therapeutic modality, and while this approach has various benefits (Natale et al., 2023), there is limited direct evidence to demonstrate an impact on occupational performance and participation. Meanwhile, other promising approaches, such as symptom-focused cognitive-behavioural therapy or energy management education, have been underutilized in kidney disease. The current development of standardized occupational performance or participation measures for nephrology clinical trials (Ju et al., 2019; Manera et al., 2020b) might ultimately help to highlight the need for an expanded set of rehabilitative services to support this patient group and other patients with complex chronic diseases (Tong et al., 2017a). Continued education and advocacy by occupational therapists regarding our role in the management of chronic kidney disease and other major noncommunicable chronic diseases is another important component of increasing access to occupational therapy services for these patients.

Our participants felt hindered in occupations spanning a broad spectrum of ICF activity and participation categories, which supports findings from the SONG prioritization initiative that occupational repertoires are idiosyncratic, and all types of occupations should be included within the patient-chosen umbrella term of “life participation” (Cheetham et al., 2022). Historically, self-care performance has received the majority of attention in the kidney disease literature that pertains to occupation, perhaps because it is the most critical for living alone. However, previous literature has also alluded to the importance that patients place on other occupations, such as travel, recreation, and work (Clarkson & Robinson, 2010; Jacobson et al., 2019), and there is extensive literature to show that these occupations are critical to quality of life (Brajša-Žganec et al., 2011, p. 2017; Lloyd & Auld, 2002; Mukherjee, n.d.; Ngamaba et al., 2023). In our study, going out for a meal or attending a movie, concert, or performance were the most common tasks people on hemodialysis felt they had insufficient energy in which to participate. This finding could be attributed to the energy required to participate in community excursions, or a perceived need among participants to reserve their energy for occupations deemed essential (e.g., traveling to dialysis, showering, or grocery shopping), the latter which were less frequently chosen as priorities by our participants. An inability to participate in community-based leisure occupations can have negative implications that include poor mental health, community disengagement, and social isolation (Brajša-Žganec et al., 2011, p. 2017; Lloyd & Auld, 2002; Toepoel, 2013; Yoshida et al., 2021). Thus, interventions are needed that teach ways to conserve, allocate, and prioritize energy to support occupational balance. Our study highlights the value of capturing a spectrum of occupational domains when studying, measuring, and addressing occupational performance and participation in people with complex chronic diseases, to ensure patients receive the types and focus of support they most require.

Our finding that household management occupations were the most highly prioritized occupational goals is the first time, to our knowledge, that the top occupational priorities of people on hemodialysis have been assessed. This finding is consistent with previous studies showing that people on hemodialysis are conscious of how their disease impacts members of their family or support system, and can feel like a burden because of their performance limitations (Suri et al., 2011; White & Grenyer, 1999). It might also reflect concerns about the ability to continue to live alone in the community, given that older adults on hemodialysis are at a high risk of admission to long-term care facilities (Jassal et al., 2009; Kurella Tamura et al., 2009). With respect to specific priority tasks, almost half of the participants selected cooking as a goal, which was assigned a median importance rating of 9.5 out of 10. Cooking can be an energy-demanding activity that requires both physical and cognitive effort and is even more effortful for people on hemodialysis as they must adapt meals to meet their specialized dietary restrictions (e.g., monitoring salt and potassium in ingredients, limiting fluids, double boiling potatoes). Although people on hemodialysis typically receive dietary counselling, the priority placed on cooking by our participants suggests that further practical support (e.g., a cooking skills groups) could be beneficial for this population. However, we emphasize that further investigation in a larger sample is needed to confirm these exploratory findings.

The strengths of this study are our systematic approach to evaluating occupational performance and participation priorities and goals, which drew on two standardized occupation-based assessments to ensure a valid and comprehensive examination of these constructs. Study limitations include the small sample size and inclusion of only adults who agreed to participate in an RCT of an energy management intervention, which limits the generalizability of our findings to the broader hemodialysis population. In light of this limitation, our study should be viewed as an exploratory investigation that points to the need for further research into the occupational performance and participation experiences and priorities of people on chronic hemodialysis.

## Conclusion

Our study, while exploratory in nature, suggests people on hemodialysis who live with debilitating fatigue can experience extensive limitations in their occupational performance and participation. There is a need for novel interventions that can optimize participation in valued occupations in this population.

### Key Messages

- Kidney failure and hemodialysis treatment extensively affect occupational performance and participation.
- Household tasks are the most frequently prioritized occupations of people on hemodialysis, but there is great variation in their occupational problems and priorities that spans the domains of self-care, work and leisure.
- Occupational therapists should advocate for a greater presence on kidney care teams to help address the wide range of occupational performance and participation problems in this population.
